# The Effect of Fluid Flow Shear Stress and Substrate Stiffness on Yes-Associated Protein (YAP) Activity and Osteogenesis in Murine Osteosarcoma Cells

**DOI:** 10.3390/cancers13133128

**Published:** 2021-06-23

**Authors:** Thomas R. Coughlin, Ali Sana, Kevin Voss, Abhilash Gadi, Upal Basu-Roy, Caroline M. Curtin, Alka Mansukhani, Oran D. Kennedy

**Affiliations:** 1 Department of Orthopaedic Surgery, New York University School of Medicine, 301 East 17th Street, New York, NY 10010, USA; coughli@stevens.edu; 2 Department of Biomedical Engineering, The City College of New York, Steinman Hall, Room ST-401160 Convent Avenue, New York, NY 1003, USA; alisana155@gmail.com (A.S.); kevinjvoss@gmail.com (K.V.); 3 Department of Microbiology, New York University School of Medicine, 430-450 East 29th Street, New York, NY 10016, USA; abhilash.gadi@gmail.com (A.G.); upal.basuroy@gmail.com (U.B.-R.); alkabeemansukhani@gmail.com (A.M.); 4 Department Anatomy and Regenerative Medicine, The Royal College of Surgeons in Ireland, 123 Street Stephens Green, Dublin 2, Ireland; carolinecurtin@rcsi.ie; 5 Department of Mechanical and Manufacturing Engineering, Trinity College Dublin, Dublin 2, Ireland

**Keywords:** osteosarcoma, mechanobiology, osteogenesis, shear stress, substrate stiffness

## Abstract

**Simple Summary:**

Primary bone cancers like osteosarcoma (OS) are driven by bone cells that develop mutations, and behave in an uncontrolled manner. All bone cells, including defective ones, are sensitive to their physical environment. We tested the response of osteosarcoma cells, at the gene level, to two different kinds of mechanical stimulus, which are relevant to the tumor microenvironment. The first was fluid flowing over their surface, and the second was stiffness or rigidity of the surface beneath. In our study we found that fluid flow in particular has the ability to change the behavior of OS cancer cells, and could potentially be used to reduce their harmful effects.

**Abstract:**

Osteosarcoma (OS) is an aggressive bone cancer originating in the mesenchymal lineage. Prognosis for metastatic disease is poor, with a mortality rate of approximately 40%; OS is an aggressive disease for which new treatments are needed. All bone cells are sensitive to their mechanical/physical surroundings and changes in these surroundings can affect their behavior. However, it is not well understood how OS cells specifically respond to fluid movement, or substrate stiffness—two stimuli of relevance in the tumor microenvironment. We used cells from spontaneous OS tumors in a mouse engineered to have a bone-specific knockout of pRb-1 and p53 in the osteoblast lineage. We silenced Sox2 (which regulates YAP) and tested the effect of fluid flow shear stress (FFSS) and substrate stiffness on YAP expression/activity—which was significantly reduced by loss of Sox2, but that effect was reversed by FFSS but not by substrate stiffness. Osteogenic gene expression was also reduced in the absence of Sox2 but again this was reversed by FFSS and remained largely unaffected by substrate stiffness. Thus we described the effect of two distinct stimuli on the mechanosensory and osteogenic profiles of OS cells. Taken together, these data suggest that modulation of fluid movement through, or stiffness levels within, OS tumors could represent a novel consideration in the development of new treatments to prevent their progression.

## 1. Introduction

Osteosarcoma (OS) is an aggressive bone cancer that originates in the osteoblast lineage and is the most common primary bone malignancy worldwide. OS tumors normally develop during growth phases in children, adolescents and young adults [1]. Typically, OS develops as a solid tumor which forms at the metaphyseal growth plate of long bones in the appendicular skeleton. The most common sites are in the distal femur, proximal tibia and humerus [2]. The annual occurrence rate of OS is less than 10 per million in the USA. However, at the time of diagnosis, tumors tend to be advanced. OS also has a high rate of metastasis, and 90% of metastases are found in the lungs [1]. Thus, prognosis for metastatic disease is generally poor, and with a mortality rate of approximately 40%, OS is an extremely aggressive disease for which new treatments are needed [3,4]. 

The early stage of any cancerous development is difficult to define precisely. However, it appears that the pathological processes of OS start when multipotent mesenchymal stem cells (MSCs) in bone marrow lose their ability to successfully complete osteogenic differentiation. This can be due to multiple mutations in regulatory pathways such as p53 and retinoblastoma (Rb), and OS tumors are common in patients with hereditary mutations in these genes [5]. Confirming the involvement of these genes in the development of OS, mice with conditional deletions in Rb and p53 develop spontaneous OS tumors. At this stage, some of these cells are termed OS cancer stem cells (CSCs), and can be identified by expression of the stem cell marker Sca-1 [6]. CSCs are thought to be a self-renewing population of cells that maintain tumor development. These CSCs then form a small but persistent subpopulation of undifferentiated cells [7]. CSCs are notoriously difficult to locate, identify and target/remove. For this reason, they are strongly implicated in disease relapse since they can persist, even following treatments/surgery, in self-renewing reservoirs [8]. Once this group is established, a period of rapid cell proliferation occurs during which a second distinct pathological subpopulation emerges [9]. Pre-osteoblasts, stuck in the early stages of differentiation, produce excessive amounts of disorganized extracellular bone matrix (osteoid). This tissue is what eventually comes to make up the bulk of the OS tumor mass. The CSC population plays a central role in driving the uncontrolled growth and activity of the pre-osteoblastic subpopulation of tumor cells, and several molecular mechanisms such as oncogene activation and tumor suppressor loss have been linked to their dysregulation [10]. Although OS CSCs can be difficult to locate and characterize, one identifiable trait is constitutively high expression of an oncogene called Yes-Associated Protein (YAP) [11]. 

YAP is a potent growth promoter involved in healthy growth and development of many tissues, including bone [12], but is also reactivated and heavily involved in skeletal tumorigenicity. YAP activity is kept in check by the tumor suppressive Hippo pathway [13,14]. YAP activity is also controlled, at a transcriptional level, by Sox2, which is well known for its role in maintaining stemness. Sox2 is one of a suite of genes that regulate the osteoblast lineage from its multipotent MSC lineage, and thus also has a role in cancers that originate from dysregulation in this lineage [15]. An important mechanism that links Sox2 activity with CSCs is its role in regulating the tumor suppressive Hippo pathway [15]. Specifically, Sox2 represses two Hippo activators, Nf2 and WWC1, thereby leading to YAP stabilization downstream and its ability to effect transcription of YAP target genes in the nucleus. Thus, inhibition of Sox2 can increase Hippo activity and restrain YAP by phosphorylation and nuclear exclusion and thus reduce tumorigenicity [16,17]. YAP is potently oncogenic in several tumor types [11,13,17] and we have found that YAP is overexpressed in several mouse and human OS cells lines where its nuclear expression marks a population of Sca-1^High^ multipotent CSCs [18]. YAP ablation in mouse OS cells restores osteoblast differentiation, reduces tumorigenicity and sensitizes cells to chemotherapy, suggesting that YAP expression is heavily involved in maintaining CSCs in OS [19]. In line with these findings, agents that lead to differentiation of OS CSCs cause cytoplasmic sequestration of YAP and decreased expression of its target genes [10].

Intriguingly, YAP has also been shown to be a highly mechanosensitive factor [20,21]. This is relevant for two reasons. Firstly, bone cells are well known to be highly sensitive to shear stresses created by localized interstitial fluid movements [22]. Solid tumor development has been shown to cause differential localized fluid movements, and increased interstitial pressure within the tissue [23,24]. This leads to differential fluid flow shear stresses (FFSS) throughout the tumor mass, and its local microenvironment [25,26]. Secondly, at the local tissue level, rapid and poorly controlled tumor growth results in a highly heterogeneous tissue microenvironment at the material level [27]. Native healthy bone alongside increasing amounts of poorly organized osteoid tissue form a heterogeneous and disparate substrate for tumor cell populations. Thus, knowledge of how OS cells behave in environments with differing physical/mechanical properties (e.g., FFSS/substrate stiffness), representative of the tumor microenvironment, is an important aspect of understanding disease development and progression. Our goal was to determine the effect of FFSS, and substrate stiffness on tumorigenic and osteogenic characteristics of OS cells. Specifically, we isolated primary murine OS cells, and used these two stimuli to modulate YAP activity (via Sox2) and osteogenic differentiation via bone-specific genes.

## 2. Materials and Methods

### 2.1. Isolation, Transfection and Sox2 Knockdown in mOS482 Cells

Primary osteosarcoma cells (mOS482), isolated from spontaneous OS tumors occurring in a mouse model that was engineered to have a bone-specific knockout of pRb-1 and p53, and two tumor-suppressive genes in the osteoblast lineage have been previously described [18]. Qualitative histological evaluation of the tumor mass, on standard formalin fixed paraffin embedded (FFPE) samples, was also carried out to assess tumor tissue composition and structure. In order to study the role of Sox2 and YAP, and the relationship between the two in tumorigenesis and osteoblast function, msOS482 cells were transduced with either non-targeting scrambled short hairpin RNA (Scr) or targeted shRNA for Sox2 (shSox2) using retroviral transfection. Standard immunocytochemistry (ICC) methods were used to evaluate transfection efficiency. Briefly, cells were washed, fixed (4% paraformaldehyde at 37 °C) and permeabilized (0.1% Triton X-100) in PBS, and further blocked for 30 min with 2% BSA/PBS. Then, cells were incubated with a primary polyclonal antibody against Sox2 (48–1400, Thermo Fisher, Branchburg, NJ, USA) in 1:200 dilution for 1 h. After two washes with PBS, cells were incubated with secondary antibody at 1:200 (A11034, Alexa Fluor^®^ 488, Thermo Fisher, Branchburg, NJ, USA), phalloidin (A22283, Alexa Fluor^®^ 546, Thermo Fisher, Branchburg, NJ, USA) at 1:500 dilution, and DAPI (P36935, Thermo Fisher, Branchburg, NJ, USA) for nuclear staining. Finally, imaging was carried out with a Nikon Ti-Eclipse (Nikon, Japan) microscope at 40X magnification.

### 2.2. Fluid Flow Shear Stress (FFSS)

For FFSS studies, mOS482 cells were plated on collagen type I coated microscope slides at a density of 1 × 10^5^ cells/slide and cultured in high-glucose MEM media (Sigma Aldrich, Carlsbad, CA, USA) for 24 h. Cells were then placed in a commercial fluid flow system (Flexcell Corp. Int., Burlington, NC, USA), which subjected them to a continuous laminar fluid flow regime, at a calculated shear stress level (at cell surface) of 1 kPa. We, and others, have previously found this to be a physiologically relevant mechano-stimulatory shear stress level [28]. The system was maintained in high-glucose MEM media with standard penicillin/streptomycin (Sigma Aldrich, Carlsbad, CA, USA) for 2 h. The cells were also treated with hygromycin (Sigma Aldrich, Carlsbad, CA, USA) in all experiments to ensure retention of vector cargo. Control cells were plated on slides and maintained in the same media and conditions but not subjected to flow. Cells were then collected and processed for subsequent analyses. Firstly, the intracellular location of YAP was determined using ICC and image analyses. ICC was carried out as described above—cells were washed, fixed and permeabilized, and then blocked with 2% BSA/PBS. Cells were then incubated with a primary monoclonal mouse antibody against YAP-1 (10413M1, Sigma Aldrich, Carlsbad, CA USA) in 1:400 dilution for 1 h. After two washes with PBS, cells were incubated for 1 h with goat-anti-mouse secondary antibody at 1:200 (20691, Thermo Fisher, Branchburg, NJ, USA) and then imaged as before. Then, to assess its activity as well as location, gene expression of direct downstream targets of YAP (cysteine-rich angiogenic inducer 61 (CYR61)), connective tissue growth factor (CTGF), and brain-derived neurotrophic factors (BDNF)) were also measured using standard qPCR methods. To assess the osteogenic status of the cells in each experimental group, standard gene expression studies were also carried out to assess runx2, osterix, osteocalcin and osteoprotegrin (OPG) levels. For these, and all subsequent qPCR procedures in this study, total RNA was extracted using the RNeasy mini kit (Qiagen, CA, USA) and treated with DNase following manufacturer protocols. Then, 0.5 μg of purified RNA was reverse transcribed at 42 °C for 65 min using SuperScript II RT and Oligo(dT) as a primer in a final volume of 20 μL and 2 μL of sample was used as a template for amplification using gene-specific primers sets. RT-PCR was carried out on a Light Cycler Instrument using the DNA Master SYBR Green I dye intercalation assay with B-actin used as a normalization control. 

### 2.3. Polyacrylamide (PA) Substrate Fabrication and Mechanical Testing

Polyacrylamide (PA) hydrogels were used in these studies as substrates with easily modifiable stiffness. Stiffness levels of approximately 1 and 60 kPa were used as representative ‘soft’ and ‘stiff’ substrates, respectively. These values were chosen to reflect physiological stiffness levels at either end of the ECM tissue spectrum in OS [6]. Furthermore, these values have also been used in other studies to represent similar disparities in tissue stiffnesses [20]. Briefly, hydrogel fabrication was carried out by coating sterilized glass slides with 0.4 mL of 0.1 M NaOH; and glass coverslips first with 0.2 mL of 3-aminopropyltrimethoxysilane (3-APTMS) and then with 1 ml of 0.5% glutaraldehyde. At each stage, incubation/rinsing was carried out as appropriate. Ammonium persulfate, 40% acrylamide and 2% bis-acrylamide were sterilized through 0.2 µm filters. Acrylamide, bisacrylamide, TEMED and PBS were combined to achieve the intended stiffness. Hydrogel solution was delivered onto treated coverslips, then a second coverslip, treated with SurfaSil to siliconize the glass surface, was used to cover the construct which was then allowed to polymerize. For crosslinking, Sulfo-SANPAH was delivered to each hydrogel and cured using UV light (at 320–365 nm) for 10 min. Hydrogels were rinsed and 0.1 mg/mL collagen in HEPES was applied to each hydrogel before overnight incubation. Hydrogels were then maintained for 3 days to allow for swelling to occur and equilibrium to be reached. For mechanical analysis cylindrical hydrogel samples (15 mm diameter; 5 mm height) were formed using standard molds. Mechanical testing was performed using a Bose Electroforce 3200 with impermeable circular platens. Samples were secured between platens and tested in compression under displacement control at a rate of 0.001 m/sec and stiffness values were calculated at 10% strain [29]. System-based WinTest software was used to record test data which was then exported to a custom Matlab script for processing and analyses. 

### 2.4. Cell Culture on PA Substrates

Cell culture studies on PA substrates were carried out in standard conditions, as described above, for 10 days using supplemented MEM media. Hydrogels were fabricated and treated as before. Firstly, the effect of substrate stiffness on mOS482 cell proliferation was determined. Using a standard CCK-8 absorbance assay, cell growth on each substrate was measured using a hemocytometer every 24 h. Then ICC was used as before to measure the intracellular location of YAP. Cells from each substrate group were fixed, permeabilized and incubated with a monoclonal mouse antibody against YAP (and goat-anti-mouse secondary) in BSA solution as described above. In order to assess the activity of the YAP protein, in addition to its location, as before, gene expression of downstream targets was measured via qRT-PCR of its direct downstream targets (CYR61, CTGF and BDNF), in order to assess tumorigenic activity. Finally, osteogenic activity was assessed by gene expression of the same suite of bone-specific cells as described above (i.e., runx2, osterix, osteocalcin and OPG).

### 2.5. Statistical Analyses

Data are expressed as mean ± standard deviation (SD) of results from independent experiments. Where the assumption of normally distribute data was met, the unpaired two-tailed Student’s t-test was used, where that assumption could not be made Mann–Whitney tests were used. A difference was taken as statistically significant when the *p*-value was at least ≤0.05; specific *p*-values are shown in each figure. 

## 3. Results

### 3.1. Isolation, Transfection and Sox2 Silencing in mOS482 Cells

**Figure 1:**
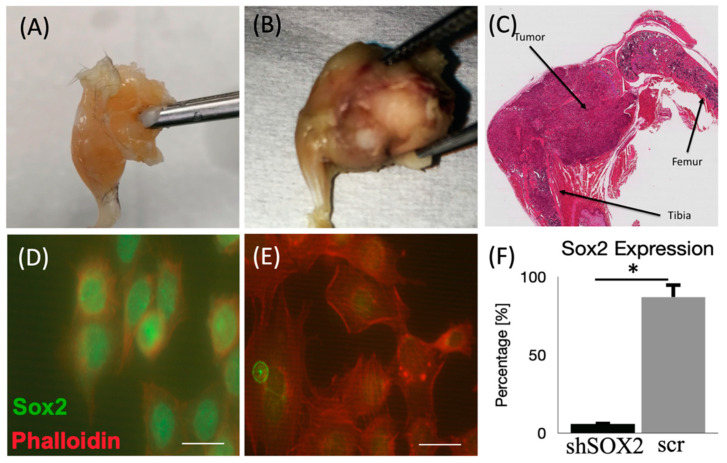
Spontaneous primary murine osteosarcoma and verification of Sox2 knockdown. Gross dissection of knee joint from (**A**) control mouse hindlimb and (**B**) Rb1 and p53 knockout mouse with significant OS tumor present. (**C**) Sagittal section through OS tumor hindlimb stained with standard H&E. (**D**) Immunofluorescence image of Sox2 (green) and actin (red) in (**D**) scr and (**E**) shSox2 treated cells; scale bar = 20μm. (**F**) Quantification of Sox2 positive cells in scr Vs shSox2; mean + SD; * *p* < 0.001; *p* value from Student’s *t*-test for statistical significance.

Gross morphology and qualitative histology confirmed the presence of a significant tumor mass in the mouse proximal tibial, likely with its origin in the growth plate. Tumor mass was relatively large and was a viable and rich source of primary OS cells (Figure 1A–C). mOS482 cells were successfully isolated from these tumor tissues and Sox2 was effectively knocked down using shRNA. Immunocytochemistry confirmed reduction of Sox2 in shSox2 cells compared to controls (Figure 1D–F). 

### 3.2. Fluid Flow Shear Stress (FFSS) Studies

**Figure 2:**
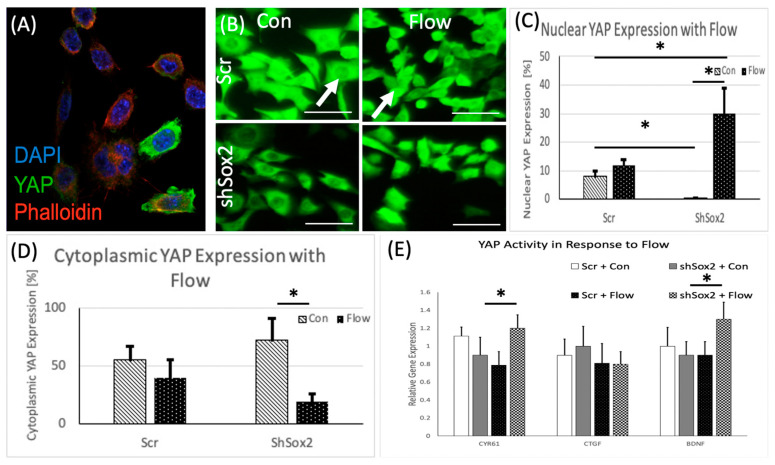
Evaluation of YAP localization and activity as a function of fluid flow induced shear stress (FFSS). (**A**) Representative immunofluorescence image showing staining for YAP (green), actin (red) and nuclei (blue). (**B**) Immunofluorescence images of YAP (green) staining in scr and shSox2 cells under control and FFSS conditions; scale bar = 20 μm. Quantification of (**C**) nuclear (**D**) cytoplasmic YAP localization in scr and shShox2 treated cells under control and FFSS conditions. (**E**) Gene expression data for downstream targets of YAP with FFSS * *p* < 0.05; *p* value from Mann–Whitney test for statistical significance.

Since Sox2 maintains stemness and active nuclear YAP expression in OS cells, we examined the effect of mechanical stress on cells expressing high or low Sox2 and examined YAP intracellular localization using confocal microscopy (Figure 2A). The percentage of nuclear vs. cytoplasmic YAP expressing cells was assessed with and without FFSS. The data showed that nuclear YAP was decreased in the absence of Sox2 alone (in the shSox2 cells), but this trend was reversed with the application of FFSS to shSox2 cells (Figure 2B–D). The expression levels of downstream targets of YAP (CYR61, CTGF and BDNF) were also assessed to determine its intracellular activity, in addition to subcellular location. These data showed that FFSS significantly increased expression of CYR61 and BDNF in shSox2 cells, while no significant changes were observed in control groups (Figure 2E). 

**Figure 3:**
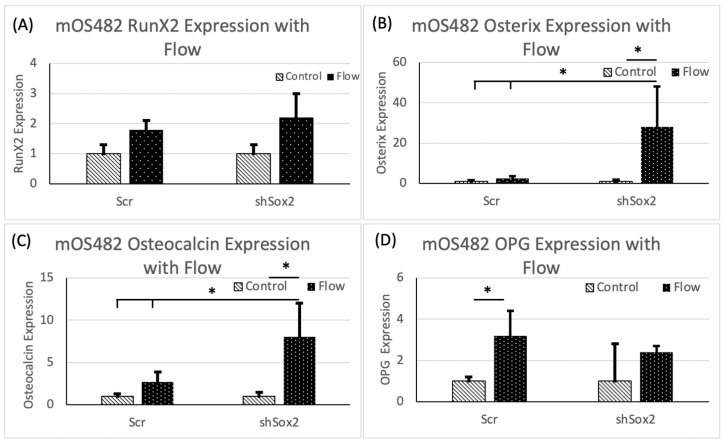
Evaluation of osteogenic gene expression in scr and shSox2 treated cells as a function of FFSS. Quantification of (**A**) Runx2 (**B**) Osterix (**C**) Osteocalcin and (**D**) OPG expression in scr and shSox2 treated cells under control and FFSS conditions * *p* < 0.01; *p* value from Mann–Whitney test for statistical significance.

Osteogenic gene expression was also increased with FFSS, an effect that was more pronounced in shSox2 cells with decreased Sox2 levels. Runx2 expression appeared unchanged by decrease in Sox2 in the shSox2 cells compared to control cells (scr) but was increased by FFSS in both groups (Figure 3A), while osterix and osteocalcin expression was significantly increased by FFSS only in the shSox2 cells (Figure 3B,C). In contrast, OPG expression increased with FFSS in scr cells, but not in shSox2 cells (Figure 3D). 

### 3.3. Substrate Stiffness Studies

**Figure 4:**
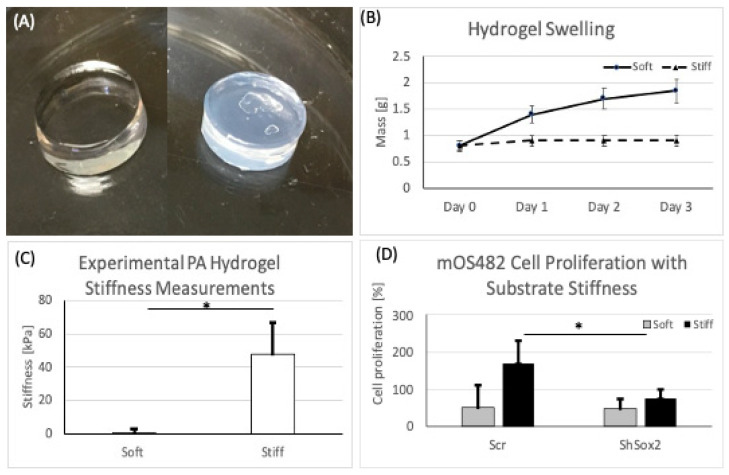
Physical characterization of PA hydrogels and cell proliferation. (**A**) Representative images of soft and stiff PA hydrogels before testing. (**B**) Swelling properties of PA hydrogels over 3 days. (**C**) PA hydrogel stiffness measurements. (**D**) OS cell proliferation on soft and stiff substrates. * *p* < 0.01; *p* value from Mann–Whitney test for statistical significance.

**Figure 5:**
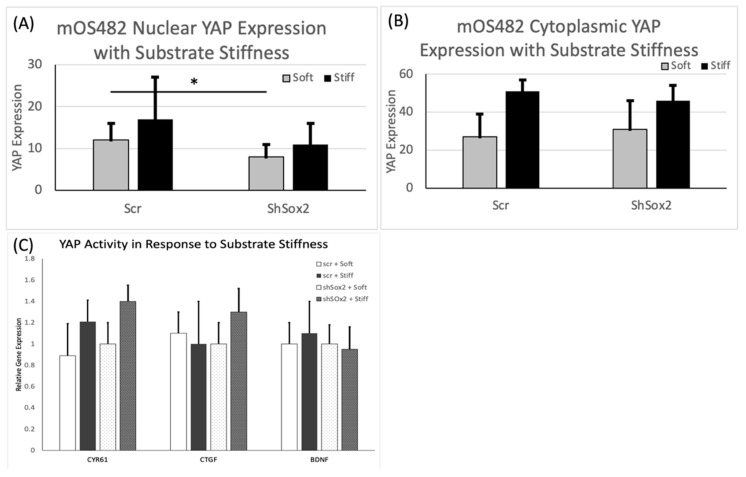
Evaluation of YAP expression, and its downstream targets, in scr and shSox2 cells as a function of substrate stiffness. Quantification of (**A**) nuclear and (**B**) cytoplasmic YAP protein expression in scr and shSox2 on soft and stiff substrates. Relative expression of downstream YAP targets in (**C**) scr and shSox2 cells with substrate stiffness * *p* < 0.05; *p* value from Mann–Whitney test for statistical significance.

**Figure 6:**
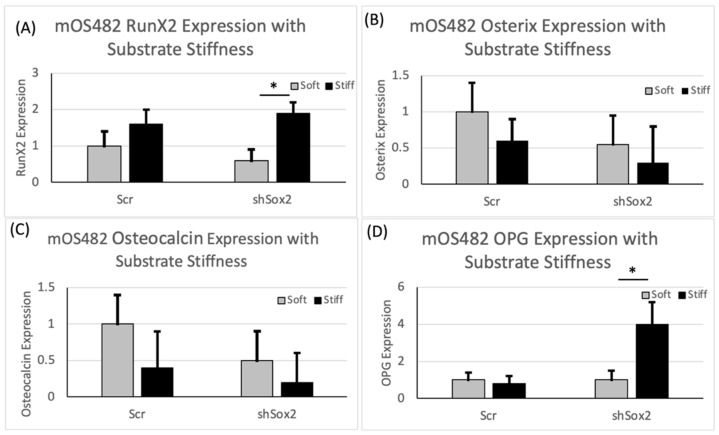
Evaluation of osteogenic gene expression in scr and shSox2 cells as a function of substrate stiffness. Quantification of (**A**) Runx2 (**B**) Osterix (**C**) Osteocalcin and (**D**) OPG expression in scr and shShox2 cells with substrate stiffness. * *p* < 0.01; *p* value from Mann–Whitney test for statistical significance.

Polyacrylamide (PA) hydrogels were reproducibly fabricated and characterized for these studies (Figure 4A). Stiffness values of 1 and 60 kPa, for ‘soft’ and ‘stiff’ groups respectively were chosen to represent relevant OS tissue properties. Mechanical testing, following equilibration (Figure 4B), confirmed these average values in our two groups (Figure 4C). Next, the effect of substrate stiffness on cell proliferation showed that increased stiffness resulted in increased cell proliferation in scr cells, but this effect was diminished in the absence of Sox2 (Figure 4D). Then, the levels of nuclear expression and cytoplasmic expression of YAP were determined on soft and stiff substrates. These data showed that nuclear YAP expression was not appreciably changed by stiffness, but that downregulation of Sox2 reduced levels of nuclear YAP on soft substrates (Figure 5A). The expression levels of downstream targets of YAP (CYR61, CTGF and BDNF) were assessed to determine its intracellular activity, in addition to location, which was unchanged by substrate stiffness in both cell groups (Figure 5C). Runx2 gene expression increased with stiffness in cells with shSox2 (Figure 6A) while expression of osterix and osteocalcin decreased slightly in both groups (Figure 6B,C). In contrast, OPG expression was significantly increased with stiffness in shSox2 treatment but was unchanged in scr groups (Figure 6D). 

## 4. Discussion

Osteosarcoma (OS) tumors account for 20% of all bone tumors and approximately 5% of all pediatric tumors, and are a highly metastatic form of malignancy. The treatment options for OS are usually surgical resection, and/or either high-dose methotrexate, doxorubicin or cisplatin—these options have not changed in more than 4 decades and 5-year survival rates remain between 20% and 30% for advanced disease. The cell type at the center of this pathology is the bone marrow-derived mesenchymal stem cell (MSC). A malfunction/mutation in genes such as p53 or Rb, which are central to cell-cycle regulation, leads to genomic instability and converts them to CSCs, that maintain stemness features and prevent proper differentiation from occurring in these cells. Once a population becomes established, it drives proliferation and activity of pre-osteoblastic cells—which ultimately proceed to form tumor masses. These OS cells have high expression of the transcriptional regulators Sox2 and Yes-Associated Protein 1 (YAP). Intriguingly, YAP (along with its paralogue TAZ) have recently been found to have a key role in many aspects of bone development, homeostasis and repair [30,31]. These transcription factors, and other upstream components of the Hippo pathway that regulate their activity, have also been directly linked with osteosarcoma [31,32]. Furthermore, these factors have also been shown to be sensitive to their physical/mechanical environments—however, whether and how this affects cell fate or disease progression remains unknown.

Previous work has shown that Sox2 marks and maintains cancer stem cells in a variety of cancers, including tumors of epithelial, neural and mesenchymal origin. This reflects not only their stem-like nature, since other stem cell transcription factors (for example, Oct4) are not expressed in osteosarcoma cells. It is therefore of great importance to understand not only how the expression of Sox2 is maintained or activated in OS, but also the mechanisms by which its expression influences disease dynamics and progression. Our data show we successfully knocked down Sox2 expression in cells harvested from primary murine OS tumors. Sox2 is known to bind the YAP promoter in osteoprogenitors as well as osteosarcoma cells, and can increase YAP activity in those cell types. Accordingly, we found that the expression of nuclear YAP was reduced following shSox2 treatment. This is potentially due to upregulation of Nf2 and WWC1 (both of which are crucial Hippo pathway activators), which have been shown to increase phosphorylation and cytoplasmic retention of YAP. However, it was intriguing to note that the application of FFSS was able to reverse this response and significantly increase nuclear YAP following treatment, but did not have this effect in controls. The expression levels of downstream targets of YAP (CYR61, BDNF) were also decreased in absence of Sox2, but then increased by FFSS, which suggests YAP’s location, as well as its activity, is influenced by this stimulus. Osteogenic gene expression was also altered by FFSS, for cells with and without Sox2. Runx2 expression increased in response to flow in both groups, while osterix and osteocalcin expression increased significantly only in shSox2 cells in which Sox2 was depleted. This suggests that FFSS can contribute to the restoration of normal osteogenesis in these cells. It may be that the mechanism behind this process is related to the well-known role of FFSS in the terminal differentiation of healthy osteoblasts into osteocytes in the mineralizing lacuna–canalicular system. Here, FFSS is the most significant physical/mechanical force that the local cell population experiences. In contrast, OPG expression increased with FFSS in the presence of Sox2, but not in its absence. These data demonstrate that OS cells are mechanosensitive and that mechanical signals generated by FFSS can increase the expression of osteogenic genes, and may direct these cells back towards a normal differentiation program. 

Soft substrates have been shown to prompt MSCs down an adipogenic lineage, whereas stiffer ones result in progression towards an osteogenic one [14]. This differential effect is reflected in nuclear localization of YAP where stiffness appears to be a cue for osteoblastic differentiation [14]. These studies highlight YAP as a mechanosensory factor and we explored whether the same would be true in OS cells due to their similar mesenchymal origin. Previous work has shown that osteosarcoma cells exposed to increased levels of stress can develop CSC characteristics, as compared to colon (25 kPa) and breast (5 kPa) cancer cells. This suggests that the organ of origin is a significant influence on the intrinsic mechanosensory characteristics of a given type of tumor cell. There is accumulating evidence that the transcriptional coactivators YAP and WWTR1(TAZ) that are downstream of the Hippo signaling pathway are exquisitely sensitive to substrate stiffness, and physical stress. In line with these concepts, our data show that cell proliferation increases on stiffer substrates in the presence of Sox2, but that this feature is lost in the shSox2 cells, indicating the importance of the Sox2/YAP axis in the proliferative effect. Interestingly, we found that nuclear YAP nuclear expression was significantly reduced on soft substrates in the absence of Sox2, and appeared to increase with stiffness. The trend in the presence of Sox2, while not significant, remained on stiff substrates. Similarly, expression of downstream markers of YAP activity in these cells were not significantly different on either substrate, in either cell type, but the trend suggested that YAP activity may be rescued by stiffness. Despite this, osteogenic gene expression was altered by substrate stiffness, for cells with and without Sox2. As was the case with FFSS, Runx2 expression increased with increasing stiffness, but just in the absence of SOX2. In contrast to FFSS stimulus, stiffer substrates seemed to reduce expression of osterix and osteocalcin but these changes did not reach significance. This suggests that substrate stiffness does not have the same potential to prompt a full return to the osteogenic pathway as FFSS. In contrast to FFSS, OPG expression was increased with stiffness in the absence of Sox2. These data reinforce the idea that OS cells are mechanosensitive and suggest that physical stimulus generated by stiffer substrates can alter the expression of osteogenic genes, but in a potentially less robust manner than FFSS. In an OS tumor mass, increased stiffness could contribute to maintaining the cells in an undifferentiated state by YAP induction. In line with this, increased stiffness has been reported in other tumor microenvironments to stimulate pro-cancerous properties. In line with this, pro-osteogenic genes did not appear to be affected by stiffness in our study. A different trend was observed in the presence of flow where increased FFSS tended to increase pro-osteogenic gene expression. It could be speculated that in a tumor setting, FFSS effects could overcome increased YAP to induce osteogenesis. Thus, these data could have implications for biophysical forces in tumor development and cancer progression.

Sox2, and thus YAP, serve as key regulators in MSC development in health and disease. YAP in particular has been linked in recent years to development of primary bone cancers (and metastases) when inappropriately activated. Thus, this transcription factor is being viewed as a promising therapeutic target for the treatment of bone cancers. However, no compounds directly targeting it have yet been identified. This may in part be due to the extremely large interaction surface of YAP with TEAD, which small molecules may find difficult to break [31]. In contrast, several small molecules binding to the YAP/TAZ interaction surface of TEAD are already in development. Taken together, this highlights the importance of this signaling pathway, and also expanding our understanding of how it responds to changes in its mechanical and physical environment.

## 5. Conclusions

In conclusion, we have described the effect of two distinct physical stimuli (FFSS and substrate stiffness), which are of relevance to OS tumors, on the mechanosensory and osteogenic profiles of murine OS cells. YAP expression, which is highly sensitive to mechanical stimulus, is also under control of Sox2, and the lack of this key regulator significantly influenced the response to mechanical stimulus—especially FFSS. Taken together these data suggest that modulating fluid movement through, or stiffness levels within, OS tumors could augment their course of progression.

## Data Availability

Data are contained within the article.
